# Psychosocial Climate as Antecedent for Resources to Manage Emotional Demands at Work

**DOI:** 10.3390/ijerph22010064

**Published:** 2025-01-06

**Authors:** Lars Peter Andersen, Dorte Ruby Andersen, Jesper Pihl-Thingvad

**Affiliations:** 1 Danish Ramazzini Centre, Department of Occupational Medicine, Gødstrup Hospital, 7400 Herning, Denmark; doteader@rm.dk; 2 Department of Occupational and Environmental Medicine, Odense University Hospital, 5000 Odense, Denmark; jesper.pihl-thingvad@rsyd.dk

**Keywords:** psychosocial safety climate, emotional demands, practical strategies

## Abstract

Background: Effectively managing emotional demands in the workplace is crucial in professions such as healthcare, education, and social work. Recent studies indicate that the psychosocial safety climate within an organization can significantly mitigate the negative effects associated with high emotional demands. Method: This study examines whether psychosocial safety climate is associated with six practical strategies for managing emotional demands that have previously been found to be associated with less burnout. It utilizes longitudinal data from 1457 participants across 129 different workplaces in various emotionally demanding professions. The associations were analyzed using linear regression and Poisson regression techniques. All analyses incorporated cluster-robust standard errors to account for potential dependencies arising from respondents sharing the same workplace. Results: The results indicate that higher levels of psychosocial safety climate are significantly associated with the availability of practical strategies. Furthermore, the incidence rate of receiving multiple types of support increases significantly for each point increase in psychosocial safety. Additionally, these relationships remain consistent even under high work demands. Conclusions: The results underscore the importance of fostering a psychosocial safety climate and increasing the availability of practical strategies to manage emotional demands in the workplace. This approach may safeguard employee well-being in high-demand professions.

## 1. Introduction

### 1.1. Emotional Demands at Work

High levels of emotional demands characterize some professions, such as health care, education, and social work [1,2]. These demands often stem from the nature of the work, which frequently involves close interpersonal interactions that require both cognitive and emotional engagement [3]. The Danish Working Environment Authority defines emotional demands at work as tasks involving direct or indirect contact with individuals [4]. In professions with high emotional demands, employees must often regulate their own emotions, which can include concealing personal feelings to maintain professionalism and provide appropriate support [5,6]. Additionally, they must adapt their communication style or behavior to suit the specific needs and circumstances of the individuals they are working with [6]. Such tasks place high demands on employees in several ways; understanding, accommodating, or managing the thoughts, emotions, or behaviors of these individuals is critical for fostering effective communication and relationships.

### 1.2. Managing Emotional Demands

Meeting these expectations often requires a process known as emotional labor, defined as the regulation of emotions and emotional expressions to meet organizational or societal requirements [6]. Emotional labor encompasses behaviors such as managing feelings, displaying specific emotions, and navigating interpersonal interactions in ways that align with established feeling rules—norms that dictate how, when, and which emotions should be expressed in different contexts [6,7]. Within the framework of feeling rules, individuals employ two primary strategies known as ‘deep’ and ‘surface’ acting. ‘Deep’ acting involves generating authentic emotions or attempting to genuinely experience and express the required emotion. On the other hand, ‘surface’ acting entails concealing genuine emotions or feigning emotions that are not truly felt, all to exhibit a professionally appropriate response [6]. For employees in emotionally demanding occupations, the constant regulation of emotions poses significant challenges to their mental health and overall well-being, and managing emotional demands at work is increasingly recognized as a critical factor in maintaining employees’ well-being. In line with this, studies have found that occupations with high emotional demands place employees at an elevated risk of adverse outcomes, such as long-term sickness, absence [8,9], and poor mental health [10].

This underscores the importance of equipping organizations and employees with resources to manage emotional demands effectively, ensuring a healthier and more supportive workplace.

However, when emotional labor at work is managed on an individual level, workplaces are absolved of their responsibility to provide care, resources, and support for their employees [11]. When emotional labor is primarily left to be managed at an individual level, it implies that the responsibility for managing the emotional aspects of the job lies on the employees more than on the organizations, and the workplaces do not proactively provide the necessary resources or support to assist their employees in managing their emotional demands.

### 1.3. Conservation of Resources (COR) Theory

Hobfoll’s Conservation of Resources (COR) theory can provide a robust framework for understanding the relationship between emotional demands, organizational resources, and employee well-being. At its core, COR theory posits that individuals strive to acquire, retain, and protect resources, which are essential for coping with stress and maintaining psychological health [12]. In this theory, resources are categorized into object resources (e.g., tools for work), condition resources (e.g., employment and tenure), personal resources (e.g., self-efficacy and optimism), and energy resources (e.g., knowledge and money). Particularly relevant in this context are secondary resources, which support the preservation or acquisition of primary resources. In the workplace, secondary resources may include training programs, effective leadership, or clear communication systems. By reducing workplace ambiguities or challenges, secondary resources help maintain primary resources, thereby enhancing employees’ ability to manage high emotional demands. By addressing emotional demands at the organizational level, management can allocate secondary resources more effectively and reduce the risk of overwhelming individual employees [12]. Indeed, a meta-analytic review has shown that, within the framework of COR theory, resources in the form of both leader and coworker support are inversely associated with employees’ symptoms of burnout [13]. These findings have been corroborated by Shoman and colleagues (2021). In their systematic review, they found that social support from both coworkers and managers had a protective effect against burnout; however, the effects were small, and the evidence remains questionable [14]. Furthermore, support is measured generically in most studies, which leaves the type of support that may be most beneficial largely unexplored [14].

To manage emotional demands at the organizational level, studies have identified organizational culture and climate as moderating factors in the relationship between emotional demands and their adverse outcomes [15,16]. In the presence of a supportive or cooperative organizational climate, burnout tends to decrease, even when there is a high level of emotional demands [17,18]. It has been found that organizational resources, such as the availability of social support and role models, enhance employee resilience to emotional demands [19]. Additionally, professional supervision is described as a necessity for healthcare professionals to effectively manage emotional demands in their work [2], and informal exchanges of emotions are also described as a necessary aspect of managing high emotional demands at work [20]. More recently, a mixed-methods study by Andersen and colleagues (2024) identified six organizational-level strategies, including supervision, discussion of cases and clients directly following an emotionally difficult task, feedback on solutions to emotionally demanding work tasks, emotional venting following an emotionally demanding client or task, rotation of work tasks for temporary relief from the most demanding tasks, and general discussion on how to cope with high emotional demands to effectively reduce symptoms of burnout, over a six-month period [21]. These strategies offer structured mechanisms to help employees process emotional challenges and recover depleted resources, aligning with Hobfoll’s Conservation of Resources (COR) theory.

Focusing on the strategic and structured levels may play a key role in understanding how support measures affect burnout symptoms. The extant literature indicates that the organizational level may interact with initiatives at the individual level in the prevention of burnout. In a meta-analysis of interventions against burnout, De Simone et al. (2021) found that initiatives at the organizational level (e.g., reducing workload, implementing schedule changes, introducing structural changes, enhancing communication between organizational members, and fostering a sense of teamwork and leadership through meetings) led to a reduction in burnout [22]. However, the effects were small, and the quality of the studies was moderate to low, with a risk of bias. Furthermore, two review studies by Pijpker and colleagues (2019) and West and colleagues (2016) found that a combination of individual and organizational/structural interventions appears to have a greater effect on employee burnout than interventions targeting only one of these levels [23,24]. Both studies concluded that knowledge about how the organizational level might influence initiatives at the individual level remains unclear, highlighting the need for research to explain these potentially crucial mechanisms. Current knowledge about how burnout prevention at the organizational level may affect prevention initiatives at the individual level is thus limited, and further understanding of the organizational factors that might enhance individual-level initiatives is needed. One factor that could be particularly important is the psychosocial safety climate (hereafter PSC).

### 1.4. Psychosocial Safety Climate

PSC is a framework designed to address the psychological well-being and safety of employees within the organizational context and refers to the shared perception of how highly an organization prioritizes the psychological health and safety of its employees, as reflected in its policies, practices, and procedures [25]. In higher PSC contexts, managers are concerned for employees’ health and well-being and consequently design jobs that have manageable demands and adequate resources [25,26]. In work contexts where managers give priority to psychological health, they are concerned about worker well-being and ensure that jobs are designed to minimize psychosocial risks [27]. As expected, studies have shown that the psychosocial safety climate is negatively associated with burnout and poor mental health [27,28].

PSC is commonly perceived as a distinctive organizational climate that endeavors to establish a safe and healthy working environment conducive to the well-being of employees [29]. The assessment of PSC levels within a work unit relies on the shared perceptions of employees, considering factors such as management support and commitment to address problems or issues impacting psychological health, as well as management prioritization of psychological health over productivity goals. PSC focuses on four aspects of organizational contexts, which include management commitment, management priority, organizational communication, and organizational participation [25].

An organization characterized by a higher PSC is likely to provide sufficient job resources to enable employees to carry out their tasks and manage the demands [25]. In jobs with high emotional demands, resources will be related to the circumstances of work organization that make it more manageable for employees to manage the high emotional demands [30]. However, the precise nature of the resources required for employees to manage emotional demands needs to be specified so that managers can implement appropriate resources to make high emotional demands more manageable for the employees. This is particularly important when management wants to be proactive in addressing the consequences of emotional demands and is attentive to providing resources that can alleviate the burden on employees.

Higher levels of PSC signal an organizational commitment to safeguarding employees’ mental health, thus reducing resource depletion. Furthermore, PSC may facilitate the availability of secondary resources to prevent burnout. While research has established a general link between PSC and employee well-being, the specific mechanisms through which PSC influences the availability of resources for managing emotional strain and burnout remain underexplored. According to the Conservation of Resources (COR) theory, workplace conditions function as essential gatekeepers to resource caravan passageways [12]. Resources do not exist in isolation; rather, they operate within a dynamic social context, which is critical to their preservation or erosion [31]. PSC may serve as a critical organizational safety factor that facilitates the availability of secondary resources, such as supervision, training, and feedback, to help manage emotional demands. It functions as a safety signal, reassuring workers that seeking support from others—for instance, to address emotional demands—is both acceptable and encouraged [32].

Therefore, this study will examine if PSC is associated with the experience of the availability of resources in the form of six practical strategies to manage emotional demands. The six practical strategies are supervision, discussion of cases and clients directly following an emotionally difficult task, feedback on solutions to emotionally demanding work tasks, emotional venting following an emotionally demanding client or task, rotation of work tasks for relief from the most demanding tasks, general discussion on how to cope with high emotional demands, all of which have been identified in a previous study as resources to manage emotional demands and to protect against burnout [21]. The importance of PSC in fostering employee well-being is well-established; however, less is known about PSC’s role in enabling resources at the individual level to manage emotional strain and burnout. Addressing this gap is critical as PSC is theorized to influence workplace practices by shaping organizational norms and behaviors that prioritize psychological health.

This study will therefore investigate if PSC is associated with the workers’ experience of the availability of resources in the form of six practical strategies identified in prior research as being effective in managing emotional demands and protecting against burnout. Despite the known efficacy of these practical strategies in mitigating the negative effects of emotional demands, there is no research on how PSC facilitates or hinders the implementation of these strategies, leaving a critical gap in our understanding of effective workplace interventions. By examining the association between PSC and the availability of six practical strategies to manage emotional demands across various professions, this study aims to provide actionable insights for organizations looking to enhance employee well-being in emotionally demanding work environments.

Additionally, when examining the role of PSC, there are only a limited number of longitudinal studies and multilevel studies that have taken the potential nested nature of workplace data into account. Multilevel approaches are essential for assessing climate constructs like PSC as these are often conceptualized at the group level rather than being limited to individual workers’ experiences [33]. Finally, high work demands have previously been found to be associated with lower levels of PSC [34,35], and it is unclear whether PCS is associated with resource allocation in the context of high work demands.

Therefore, this study will examine the following questions:

1. Is the psychosocial safety climate associated with an increased level of resources in the form of the six practical strategies to enable employees to manage emotional demands at the organizational level—both cross-sectionally and longitudinally?
2. Is psychosocial safety climate associated with a higher number of practical strategies to enable employees to manage emotional demands (i.e., how many practical strategies are provided)—both cross-sectionally and longitudinally?
3. After adjusting for qualitative demands, is psychosocial safety climate associated with a higher level and number of resources in the form of the six practical strategies to enable employees to manage emotional demands—both cross-sectionally and longitudinally?

## 2. Methods

Survey data were gathered using convenience sampling, inviting employees from 129 different workplaces with high emotional demands. The workplaces were selected based on prior research that identified that employees in such settings reported high emotional demands [36]. The workplaces were contacted via email or telephone. The participants included employees from schools, including special schools, psychiatric departments, youth and family departments, job centers, crisis centers for women affected by violence, nursing homes for elderly individuals with substance abuse issues, and similar institutions. This included healthcare professionals (such as nurses, doctors, health assistants, physiotherapists, ergonomic therapists, and psychologists), social workers (including municipality unemployment counselors, and social benefit authority workers), and workers within education and pedagogy (such as school teachers, kindergarten teachers, special educators, special teachers, teacher assistants, and teacher substitutes), along with other personnel from the same organizational units (administrative and front desk workers, unskilled helpers, and consultants).

Due to GDPR regulations, we did not collect personal (personally identifiable) email addresses from each participant. Instead, an email with a link to the questionnaires was sent to the local manager. The manager then forwarded the email to the employees. The email included a link leading to the questionnaire, which explained the project and data usage. Then, upon obtaining written consent, the employees were registered in the project and directed to an internet-based survey. Due to the nature of this process, we do not know how many individuals filled out the questionnaire, and we were unable to calculate a response rate.

Survey data were collected at baseline (T1) and at follow-up after three months (T2). A 3-month period was selected as it represents an optimal balance, allowing adequate time for participants to reflect on meaningful changes while minimizing the risk of recall inaccuracies.

A total of 1457 individuals chose to paticipate, with 1094 providing responses on both baseline and follow-up questionnaires, resulting in an attrition rate of 25%.

### 2.1. Psychosocial Safety Climate

The psychosocial safety climate scale was used to measure employees’ views on organizational policies, practices, and procedures related to employee psychological well-being [29]. The PSC scale consists of 12 items and comprises four dimensions grounded in theory: how employees perceive senior management (1) engaging, (2) prioritizing, (3) communicating with, and (4) involving employees in addressing psychosocial workplace safety concerns [9,14]. However, in this study, PSC was measured by 4 items, with each item covering one of the four dimensions. A Swedish study confirmed the validity and usefulness of PSC-4 as an instrument to indicate good, fair, and poor occupational safety and health practices [37].

Each item was answered on a Likert Scale from 4 (=strongly agree) to 0 (=strongly disagree) (Cronbach’s Alpha = 0.90).

### 2.2. Practical Strategies to Manage Emotional Demands

The outcome variable was existing organizational practices used at the workplace to manage emotional demands at work. We interviewed a board of interests consisting of representatives from major Danish unions and employer organizations targeting the work environment. The interview was focused on their considerations and experience of major practices within the selected sectors in regard to managing emotional demands. This resulted in 6 major categories that describe everyday work practices, i.e., supervision, discussion of cases and clients directly following an emotionally difficult task, feedback on solutions to emotionally demanding work tasks, emotional venting following an emotionally demanding client or task, rotation of work tasks for relief from the most demanding tasks, and general discussion on how to cope with high emotional demands. Each category was formulated into a question, for instance, “To what degree does your workplace provide the possibility to discuss emotionally challenging/difficult patients, citizens, or pupils with colleagues and/or management?” and “To what degree do your workplace provide the possibility to “vent” with colleagues/management regarding emotionally demanding/difficult patients’, citizens’, or pupils’ cases?”.

Each item was answered on a Likert Scale from 0 (=to a very low degree) to 4 (=to a very high degree). For the Poisson regression analysis, each of the 6 items was recoded into only two responses. The response categories 1–4 were coded as the practical strategies were available (1), while response category 0 was coded as the practical strategies were not available (0). Subsequently, a scale ranging from 0 to 6 was developed.

### 2.3. Quantitative Demands

Quantitative demands were measured with the validated scale of quantitative demands from the Danish Psychosocial Questionnaire [38]. The Quantitative demand scale consists of 4 items, for instance, “How often does it happen that you don’t complete all your work tasks?” and “Do you have enough time for your work tasks?”.

Quantitative demands were measured on Likert scales from 1 (=never/almost never) to 5 (=always) (Cronbach’s Alpha = 0.86).

### 2.4. Covariables

Information about gender was measured as male, female, and other gender orientation; age was measured in intervals of <20 years; 21–29 years; 31–40 years; 41–50 years; 51–60 years; and >60 years. Tenure was also measured in intervals of >1 year; 1–5 years; 6–10 years; 11–15 years; 16–20 years; and >20 years. All measurements at baseline were obtained from the baseline questionnaire.

### 2.5. Sectors

Finally, we included a variable on sectors because each sector might entail context-specific characteristics related to the overall type of emotional demands. Similarly, each work area could involve psychosocial work environment factors associated with burnout that were not captured in the survey in a way that was relevant to all respondents. The 23 professional groups in the sample were recoded into four overall work areas: health professionals (nurses, doctors, health assistants, physiotherapists, ergonomic therapists, and psychologists); social workers (municipality unemployment consultants and social benefit authority workers); teaching/pedagogy (including school teachers, kindergarten teachers, special educators, special teachers, teacher assistants, and teacher substitutes); and other professions (administrative and front desk workers, unskilled helpers, and consultants).

### 2.6. Analyses

We chose to use both linear regression analyses and Poisson regression analyses because linear regressions can provide detailed information about small changes in the index depending on changes in PSC whereas the Poisson regression analyzes the number of the practical strategies depending on changes in PSC and thus illustrates how the number of practical strategies varies with different levels of PSC. Employing both approaches in regression analysis can offer a comprehensive understanding of how PSC influences the practical strategies in various ways—ranging from minor continuous changes to changes in the actual number of practical strategies.

Therefore, our analyses focused on two outcomes: practical strategies as an index and the total number of practical strategies. The former is viewed as a continuous scale and was analyzed using multiple linear regression analyses, whereas the latter was considered a count outcome and was analyzed using Poisson regression. Both outcomes were analyzed cross-sectionally (all outcomes and predictors measured at T1) and longitudinally (outcome measured at T2 and predictors measured at T1).

Both cross-sectional and longitudinal analyses were performed in three steps. First, a crude model was estimated, including median-centered psychosocial safety climate as a predictor as well as baseline levels of the predicted outcome for longitudinal analyses. Second, gender (reference: female) and age groups (reference: 41–50 years old) were added as covariates. Lastly, to see if work demands influence the effect of psychosocial safety climate on a given outcome, median-centered work demands were added to the model.

All analyses included cluster-robust standard errors, correcting for possible dependence resulting from respondents sharing the same workplace. For multiple regression models, homoscedasticity and normality of residuals were inspected using scatterplots and QQ plots, whereas linearity of psychosocial safety climate was tested using natural cubic splines with 5 knots. For Poisson regression models, model fit was visually inspected using a scatterplot of predicted versus observed counts and tested using Pearson’s goodness-of-fit statistic.

All analyses were performed in Stata 17 (Stata Corporation, College Station, TX, USA).

## 3. Results

As shown in Table 1, most participants were women, and more than half of the participants were between 31 and 50 years old. Most participants have between 1 and 10 years of experience in their current workplace.

As shown in Table 2, about half of the employees report that the workplace provides them with all six practical strategies to manage emotional demands; 60.9% at T1 and 53.2% at T2 reported that the workplace provides them with all six practical strategies to manage emotional demands.

Table 3 shows that when adjusting for gender, age, experience in the current workplace, and even work demands, for each point increase in psychosocial safety climate at T1, the practical strategies measures at T2 will increase significantly. Furthermore, it can be seen from Table 3 that when adjusting for gender, age, experience, and work demands in the current workplace, for each point increase in psychosocial safety climate at T1, the practical strategies at T2 will increase significantly.

Finally, Table 4 shows that when adjusting for gender, age, experience, and work demands in the current workplace, the incident rate for receiving multiple types of support at T2 increases significantly for each point increase in psychosocial safety at T1. 

## 4. Discussion

This study found that PSC was associated with an increased number of the provided practical strategies to manage emotional demands at the organizational level both cross-sectionally and longitudinally. This is particularly relevant when examining small changes in the index of the practical strategies provided as they were related to variations in PSC (using linear regression) and when analyzing how the number of practical strategies differs at various levels of PSC (using Poisson regression). The former analysis shows that when employees feel safer in their work environment (higher PSC), the practical strategies are improved. The latter analysis shows that as employees perceive a safer psychosocial environment at work, their chances of receiving different types of support increase. Both cross-sectional and longitudinal analyses showed that when employees feel that their supervisors are concerned for their health and well-being and, consequently, design jobs that have adequate resources, they experience an increased number of practical strategies provided to manage emotional demands.

Finally, the associations between psychosocial safety and the six practical strategies were statically significant, irrespective of work demands. This means that the relationships observed are robust and not affected by the level of workload employees experience. In other words, the results continue to hold true whether or not employees are facing high levels of workload.

Although the effect of PSC on practical strategies is modest, it can still hold practical significance. For instance, if small improvements in PSC lead to significant long-term gains, implementing changes may still be valuable. Furthermore, the relationship may become more pronounced in future longitudinal studies with a longer follow-up period. Thus, these findings represent a first step toward understanding the association between PSC and practical strategies to manage emotional demands, even if the effect is not large in the current study.

The results have expanded the understanding of how PSC might be associated with a reduction in emotional demands, as found in other studies [39,40]. Although associations between PSC and reduced demands have been established, the mechanisms by which PSC influences a reduction in emotional demands, as well as the specific resources that should be prioritized for enhancement, were unclear. For instance, the study by Biron et al. [39] demonstrated that PSC at T1 was associated with perceived lower psychological demands at T2; nevertheless, the mechanism underlying the connection between PSC and lower perceived demands remains unresolved. Furthermore, the study by Dollard et al., 2010 found a top-down effect of PSC on lower-level work entities, with PSC predicting changes in skill discretion, work pressure, and emotional demands over time [25]. However, the mechanisms connecting PSC with, for instance, emotional demands were unclear. This present study has expanded this understanding by finding a significant association between PSC and the availability of six practical strategies to manage emotional demands. The findings are important because they provide new insights into how PSC influences workplace dynamics, particularly by highlighting the role of PSC in the availability of practical strategies for managing emotional demands. This advances the understanding of PSC and offers actionable guidance for organizations seeking to foster healthier work environments and reduce emotional strain on employees. Additionally, the findings are important because emotional demands such as dealing with difficult customers, stressful interactions, or emotionally taxing tasks can pose serious risks to employees’ health, safety, and well-being [8,41,42].

Greenfield (2020) emphasized that job-related emotional challenges are often associated with health and safety issues [43]. By identifying the practical strategies tied to PSC, the results from this study underlines the importance of PSC as a possible prerequisite for practical strategies to protect against the strain of jobs with high emotional demands. PSC may act as a critical organizational safety factor that enhances the availability of secondary resources [32,40]. These findings deepen the understanding of the role of PSC not only in promoting a positive work environment but also in promoting actionable ways to safeguard workers’ emotional resilience. Furthermore, the statistically significant associations between PSC and the six practical strategies, irrespective of work demands, are promising and reinforce the robustness of the findings.

The practical implications of this study indicate that to enhance the psychosocial work environment, it is essential for management to promote PSC. PSC is frequently regarded as a management aspect capable of assisting employees in minimizing job demands while enhancing job resources. Companies implementing PSC may be able to design better workload management procedures and supply job resources [44,45]. This can be accomplished by making efforts to educate supervisors on the significance of a positive PSC and by developing management practices that actively reduce the risk of burnout. Management has the power and position to enhance employees’ opportunities and use practical strategies to manage emotional demands. Managers possess the power and resources to implement these changes [25], and their commitment is important to any workplace policies targeting employees’ well-being [39,46].

It may also be essential to establish clear procedures and policies that foster a positive psychosocial climate. This may include guidelines for managing high emotional demands. Finally, a supportive work environment should be cultivated so that employees feel safe reporting stress and mental health challenges. Encouraging open dialogue between employees and management can help identify and address potential risks before they escalate into serious issues. By implementing these recommendations, workplaces can improve the psychosocial work environment. This enhancement will not only benefit employee well-being but also increase overall workplace efficiency. However, future research should examine the optimal implementation of these strategies. The effectiveness of the six strategies may vary significantly depending on the quality and fidelity of their implementation. Additionally, it is widely recognized that understanding and assessing the effects of an intervention, for instance, implementing the six practical strategies, will require a comprehensive process evaluation [47]. Addressing the variability in strategy implementation and ensuring intervention fidelity are important areas for future research. Consequently, future studies should examine the conditions under which these strategies are most effectively implemented.

### Strengths and Limitations

The current study possesses several important strengths that enhance both the validity and generalizability of the findings. First, the inclusion of many workplaces and participants increases the statistical power and reduces the risk of random errors. Another strength of this study is its longitudinal design. By following the participants over time, one can not only capture snapshots of relationships but also examine how the association between PSC and the six practical strategies to manage emotional demands changes over time. This is particularly important as working conditions and psychological factors are rarely static; they can change in response to both internal and external influences. The longitudinal data, therefore, facilitates the investigation of causal relationships and long-term effects, providing a more robust foundation for understanding the dynamics between PSC and the six practical strategies.

Finally, this study encompasses a diverse array of workplaces, both small and large, from various regions across the country. This geographical and structural diversity enhances the external validity of this study as the results are more representative of different types of workplaces and are not limited to a specific sector or region. Furthermore, the variety of workplaces contributes to a deeper understanding of how PSC operates in different contexts, enabling the formulation of recommendations that can be applied across a wide range of workplace environments.

The limitations of this study are important to consider when interpreting the results. The use of convenience sampling may limit the generalizability of the findings. The participants may not be representative, and thus, we cannot rule out some potential selection bias, which may reduce the external validity of the findings. Furthermore, if only employees with an interest in the topic responded, this may also have introduced potential selection problems. However, it is important to note that this study did not aim to report incidents or prevalence rates within working populations. Instead, the focus was on investigating possible associations.

Second, the data were obtained solely from self-reports, which may have introduced mono-method bias due to unmeasured third variables [48]. However, it is important to recognize that factors of the psychosocial environment are also partly intrapsychic phenomena [49]. Neglecting to include the subjective experience of these phenomena would reduce the accuracy in modeling the associations between psychosocial work environment factors. Recent studies have shown that the impact of common method bias on statistical results and the acceptance or rejection of the null hypothesis is unclear [50]. It is still uncertain whether this bias has a substantial impact. To minimize this bias, validated survey scales were used, which, to some extent, reduce bias resulting from subjective factors, except for the practical strategies for managing emotional demands. While common method bias cannot be ruled out entirely, it is unlikely that these results are primarily a product of this type of bias.

A previous study established benchmarks for high and low levels of PSC, suggesting an average score of >12.0 as indicative of good OSH practices and a score of ≤8.0 as indicative of poor OSH practices on PSC-4 [37]. The fact that we did not follow this recommendation may have been a limitation of this study. However, in this study, we did not use cutoffs or dichotomize the PSC variable; instead, PSC was treated as a continuous variable, avoiding the loss of information that occurs when categorizing or dichotomizing.

## 5. Conclusions

PSC has the potential to be a pivotal intervention point for the implementation and design of high-quality employment by adopting pro-worker and robust organizational policies and practices.

The findings of this study suggest that PSC is positively associated with the number of available practical strategies aimed at managing emotional demands, as indicated by both cross-sectional and longitudinal analyses. Therefore, PSC may play a significant role in securing workers’ access to practical strategies that can protect them against the negative strain of emotional demands at work, thereby contributing to enhanced psychological well-being among employees.

We recommend that workplace safety and health organizations educate local supervisors on the critical relationship between PSC, resource availability, and burnout. PSC-focused interventions could also help normalize open discussions about psychosocial factors and convey a clear safety signal. This is particularly important as employees are more likely to utilize social support in environments with high PSC [32].

When implementing the six practical strategies in a workplace, it is essential to involve employees throughout the process. Such involvement fosters a sense of ownership among employees, which is critical for ensuring their commitment and the successful adoption of six practical strategies [51,52].

## Figures and Tables

**Table 1:** Descriptive data.

	*n* (%)
Gender	
women	1132 (77.9)
men	310 (21.3)
missing	11 (0.8)
Age	
<20	1 (0.1)
≤21–30	120 (8.3)
≤31–40	332 (22.8)
≤41–50	453 (31.2)
≤51–60	398 (27.4)
>60	140 (9.6)
missing	9 (0.6)
Seniority at the actual workplace	
≤1 years	174 (12.0)
1–5 years	554 (38.1)
6–10 years	285 (19.6)
11–15 years	210 (14.5)
16–20 years	88 (6.1)
>20 years	131 (9.0)
missing	11 (0.8)

**Table 2:** Number of practical strategies at T1 and T2.

	*n* (%)		*n* (%)
T1 Number of Practical Strategies		T2 Number of Practical Strategies	
0	24 (2.2)	0	7 (0.6)
1	24 (2.2)	1	19 (1.7)
2	31 (2.8)	2	34 (3.1)
3	42 (3.8)	3	32 (2.9)
4	97 (8.9)	4	87 (8.0)
5	177 (16.2)	5	184 (16.8)
6	666 (60.9)	6	945 (53.2)
missing	33 (3.0)	missing	149 (13.6)

**Table 3:** Associations between psychosocial safety climate at T1 and practical strategies at T1 and T2.

	Baseline									Follow-Up								
	Unadjusted			Model 1			Model 2			Unadjusted			Model 1			Model 2		
	Coefficient	*p*	95% Cl	Coefficient	*p*	95% Cl	Coefficient	*p*	95% Cl	Coefficient	*p*	95% Cl	Coefficient	*p*	95% Cl	Coefficient	*p*	95% Cl
PSC 12	0.79	0.00	0.73–0.84	0.79	0.00	0.74–0.85	0.80	0.00	0.74–0.86	0.15	0.00	0.08–0.22	0.15	0.00	0.08–0.22	0.16	0.00	0.086–0.23
	Unadjusted: Std. err. adjusted for 118 clusters																	
	Model 1: Std. err. adjusted for 118 clusters, gender, and age																	
	Model 2: Std. err. adjusted for 118 clusters, gender, age, and work demands																	

**Table 4:** IRR associations between psychosocial safety climate at T1 and practical strategies at T1 and T2.

	Baseline									Follow-Up								
	Unadjusted			Model 1			Model 2			Unadjusted			Model 1			Model 2		
	Coefficient	*p*	95% Cl	Coefficient	*p*	95% Cl	Coefficient	*p*	95% Cl	Coefficient	*p*	95% Cl	Coefficient	*p*	95% Cl	Coefficient	*p*	95% Cl
PSC 12	1.04	0.00	1.03–1.05	1. 04	0.00	1.03–1.05	1.04	0.00	1.03–1.05	1.01	0.00	1.01–1.02	1.01	0.00	1.01–1.02	1.01	0.00	1.01–1.01
	Unadjusted: Std. err. adjusted for 118 clusters																	
	Model 1: Std. err. adjusted for 118 clusters, gender, and age																	
	Model 2: Std. err. adjusted for 118 clusters, gender, age, and work demands																	

## Data Availability

To protect the privacy of participants and ensure the confidentiality of the data, the datasets used and analyzed during this study are not publicly available but can be obtained from the corresponding author upon reasonable request.
